# Shared Decision Making in Primary Care Based Depression Treatment: Communication and Decision-Making Preferences Among an Underserved Patient Population

**DOI:** 10.3389/fpsyt.2021.681165

**Published:** 2021-07-12

**Authors:** Elizabeth B. Matthews, Margot Savoy, Anuradha Paranjape, Diana Washington, Treanna Hackney, Danielle Galis, Yaara Zisman-Ilani

**Affiliations:** ^1^Graduate School of Social Service, Fordham University, New York, NY, United States; ^2^Lewis Katz School of Medicine, Temple University, Philadelphia, PA, United States; ^3^College of Public Health, Temple University, Philadelphia, PA, United States

**Keywords:** shared decision making, depression, primary care, patient preference, underserved and unserved populations

## Abstract

**Objectives:** Although depression is a significant public health issue, many individuals experiencing depressive symptoms are not effectively linked to treatment by their primary care provider, with underserved populations have disproportionately lower rates of engagement in depression care. Shared decision making (SDM) is an evidence-based health communication framework that can improve collaboration and optimize treatment for patients, but there is much unknown about how to translate SDM into primary care depression treatment among underserved communities. This study seeks to explore patients' experiences of SDM, and articulate communication and decision-making preferences among an underserved patient population receiving depression treatment in an urban, safety net primary care clinic.

**Methods:** Twenty-seven patients with a depressive disorder completed a brief, quantitative survey and an in-depth semi-structured interview. Surveys measured patient demographics and their subjective experience of SDM. Qualitative interview probed for patients' communication preferences, including ideal decision-making processes around depression care. Interviews were transcribed verbatim and analyzed using thematic analysis. Univariate statistics report quantitative findings.

**Results:** Overall qualitative and quantitative findings indicate high levels of SDM. Stigma related to depression negatively affected patients' initial attitude toward seeking treatment, and underscored the importance of patient-provider rapport. In terms of communication and decision-making preferences, patients preferred collaboration with doctors during the information sharing process, but desired control over the final, decisional outcome. Trust between patients and providers emerged as a critical precondition to effective SDM. Respondents highlighted several provider behaviors that helped facilitated such an optimal environment for SDM to occur.

**Conclusion:** Underserved patients with depression preferred taking an active role in their depression care, but looked for providers as partner in this process. Due to the stigma of depression, effective SDM first requires primary care providers to ensure that they have created a safe and trusting environment where patients are able to discuss their depression openly.

## Background and Rationale

Depression is a major public health issue, and remains a leading cause of disability worldwide (1). In the United States, about 8% of adults aged 20 and over have depression in a given 2-week period, and women are almost twice as likely than men to have had depression (2). Recent findings indicate a 3-fold increase in depression symptom prevalence in the last year due to COVID-19, with amplified effects among individuals disproportionately affected by Social Determinants of Health, such as lower socioeconomic status, those who had acute exposure to psychosocial stressors (e.g., job loss) (3), women with school age children, and individuals belonging to racial/ethnic minority groups (4, 5).

While several depression treatment options exist, including psychiatric medication, therapy, or a combination approach, many individuals experiencing depressive symptoms do not engage in any form of depression care (6). Current estimates indicate that as few as 35% of patients with new depressive episodes initiate treatment (7), and only one in five individuals receive treatment that meets minimum recommended standards care (8), with traditionally underserved populations, specifically Black, Indigenous, and People of Color (BIPOC) and economically disadvantaged groups, accessing treatment at disproportionately lower rates (9). Together, this evidence signals an urgent need to improve both access and continuity in depression treatment, particularly among underserved communities. Broadly, underserved populations are defined as minority populations or communities, or groups that experience disproportionately poorer health outcomes (10). Within this study, we use the term *underserved populations* to refer to individuals experiencing poorer outcomes related to depression specifically, which includes BIPOC individuals and lower socioeconomic backgrounds.

The need to improve depression care for this group is particularly acute within primary care settings, as many individuals experiencing symptoms of depression initially present to primary care physicians, rather than behavioral health providers (11). In addition to being the leading prescribers of antidepressant medication (12), primary care providers therefore also play a critical role in linking individuals to behavioral health care and other social services, and monitoring symptoms over time (13, 14). Consequently, the effectiveness of primary care based interventions for depression may rely on the provider's capacity to engage patients in discussions about various evidence-based treatment options in order to determine an optimal path forward. Many primary care providers report a lack of foundational knowledge and training in the treatment of common mental illnesses, including depression (15, 16), leaving them less equipped to engage in such critical conversations. This suggests that efforts to improve the quality and effectiveness of depression treatment may require increased attention to clinical practice strategies that support providers in initiating discussions about depression treatment options with their patients.

Shared decision making (SDM) is a health communication model designed to optimize treatment of chronic conditions, including depression (17), and is particularly suited for clinical scenarios, such as depression care, where multiple efficacious treatment options exist. The SDM model provides a framework for collaborative information exchange between patients and clinicians, promotes informed decision-making by encouraging discussions about treatment options, and clarifying patients' treatment preferences (18, 19). Developed as a shift away from more paternalistic models of care that privilege the authority of providers, principles underlying SDM include mutual respect, a regard for supporting self-determination, and a recognition of the patient as an expert on their own recovery (20–22). SDM is specifically recommended for use in primary care settings (23), where providers treat a diverse range of conditions.

A small number of studies have tested the impact of SDM interventions and decision aids on depression outcomes in primary care (24–27). Overall, these studies found that SDM tools increased patient involvement in care and knowledge about treatment options, but did not improve depression symptoms depression treatment initiation and adherence. Research examining SDM behaviors (28, 29) and patient-provider interactions (30, 31) during clinical visits have concluded that SDM practices are both poorly implemented and infrequent in depression care (32). In addition, these studies have primarily been conducted in rural areas and with predominantly white samples, leaving much less known about how SDM is being integrated in primary care based depression treatment among the underserved populations that are least likely to access and receive quality depression care, including BIPOC individuals. This gap is notable in light of existing studies in medicine suggesting that doctors are less likely to discuss treatment options or reasons for treatment (33) and elicit patient feedback less often (34) when the individual belongs to a racial or ethnic minority group. Self-rated SDM has also been found to be lower among individuals that are uninsured or underinsured, have lower educational attainment and are have a low socioeconomic status (35). Further, research has also indicated that patients' individual preferences around communication and decision-making vary across demographic, cultural and racial/ethnic characteristics (36, 37). Together, this suggests that current research may not accurately represent the experience of SDM among underserved populations, and that more work examining how to optimize the SDM model for individuals from diverse backgrounds are needed (38).

Alegría et al. (38) have conducted, to our knowledge, one of the few studies that has specifically examined the potential of SDM to improve the quality of behavioral health treatment among a racially and ethnically diverse individuals. This randomized controlled trial found that interventions designed to improve SDM practices increased patient ratings of service quality, but not their self-reported ratings of SDM. This suggests the potential of benefit of SDM, but signals that there is still more to learn about how to improve the experience of collaborative decision-making among this group. Further, this sample was not conducted in primary care and was not limited to those with depression diagnoses; due to the uniquely individualized process of choosing depression treatment, more explicit examination of SDM within the context of depression care is needed.

In sum, existing research has produced mixed findings about the use of SDM to optimize depression treatment, and points to the need to better understand factors that shape the patient experience in depression care, particularly among those from underserved backgrounds, including from racial/ethnic minority groups or low socioeconomic status. One consistent trend across studies is that methods to increase the patients' subjective experience of SDM are not well-understood. Within the context of depression care, little is known about how patients, particularly those from diverse backgrounds, actually prefer choices about their depression to be made. This includes desires relating to patient-provider communication, and decision-making processes, and preferences for autonomy. Efforts to increase the adoption of SDM in depression care without a thorough understanding of how patients define optimal SDM practices within this context may therefore result in the dissemination of clinical strategies that are misaligned with those that will most effectively engage patients in decisions around depression treatment. This gap is particularly problematic for underserved groups, which are among the least likely to receive optimal depression treatment. In order address dearth of information, and to inform efforts to enhance SDM in primary care based depression treatment for underserved patient populations, the purpose of the present exploratory qualitative study was to (1) examine the experiences of SDM among an underserved patient population receiving primary-care based depression treatment, including how often and in what ways they experience SDM in their care, and (2) explore communication and decision-making preferences among this group, including how effective SDM is described.

## Materials and Methods

### Design, Study Setting, and Participants

A mixed-methods study was conducted in the midst of COVID-19 pandemic between June 2020 and February 2021. Participants were recruited from primary care practices within Temple University Hospital, a large, urban safety net hospital serving one of the poorest catchment areas of Philadelphia, PA, USA. Safety net clinics, including those included in this study, refer to practices that predominantly serve patient populations that are uninsured or underinsured, economically disadvantaged, or from racial and ethnic minority backgrounds, reflecting underserved groups that have been historically underrepresented in SDM literature, and are also lease likely to receive care for depression. Consistent with safety net target populations, the patient population of Temple University Hospital is predominantly Black or African American, and more than 70% of the patients receive Medicare or Medicaid.

Eligible patients were identified using chart data abstracted from the electronic health record, and included English speaking adults (18 years or over) with a diagnosis of depressive disorder as defined by ICD-10-CM diagnostic codes F22 and F33. Using a complete list of eligible participants, a consecutive sampling approach was used to recruit patients were recruited over the phone by three bachelors level research assistants (DW, TH & DG), who were not previously known to eligible participants. As required by COVID-19 restrictions, individuals agreeing to enroll in the study participated in an interview conducted over the phone or via video conferencing. Interviews with participants were audio recorded, and lasted an average of 45 min.

A semi-structured interview guide was developed by the first and last authors (EBM & YZI). Following established frameworks (39) this was process was informed by existing knowledge, as derived from extant literature on SDM, and was piloted internally for comprehensiveness and flow. Questions include both *main themes* and *follow up prompts* (39) which are probing questions or responses designed to guide deeper understanding of phenomena of interest. Examples of the interview guide is included in Table 1. Interviews were conducted by trained research assistants (DW, TH, DG). Interviews began with a quantitative survey measuring patient's self-rated experience with SDM, and continued with a semi-structured qualitative interview guide targeting SDM preferences. Respondents received a $20 gift card for their participation. Study protocols were reviewed and approved by Temple University Institutional Review Board (protocol # 26820).

**Table 1:** Sample interview guide.

*Main Theme: Could you please describe the last important decision you and your doctor made about your depression?*
*Follow Up: As you reflect on this decision, in what ways was your doctor involved in this process?*
*Follow Up: As you reflect on this decision, in what ways were you involved in this process?*
*Follow Up: Is this the way you prefer decisions about your depression to be made? Why or why not?*
*Main Theme: If you had to make another decision like this with your doctor about your depression, how would you like them to be involved?*
*Main Theme: What kind of information do you need in order to make sure you are receiving the right care for your depression?*
*Follow Up: How do you usually get this information now?*

### Quantitative Methods

All study respondents completed a survey including demographic information (age, gender, race/ethnicity and education level) and clinical characteristics, including whether the respondent was currently prescribed antidepressants and/or enrolled in mental health services (counseling or therapy). The survey also included the SDM-Q-9-Psy, the only validated measure of patient rated SDM for mental health settings (40). The SDM-Q-9-Psy consists of nine statements rating to the respondents' perceived experience of SDM within the context of their care. Sample items include “my doctor wanted to know exactly how I wanted to be involved in making the decision,” and “my doctor told me that there are different options for treating my depression.” Respondents rate their level of agreement with each statement on a likert scale. Scores are summed and then transformed into a range from 0 to 100, with higher scores corresponding with higher levels of perceived SDM. Univariate statistics are presented for descriptive purposes, and summarize the sample's demographic composition and self-reported experiences with SDM.

### Qualitative Methods

All interviews were transcribed verbatim and uploaded into Dedoose qualitative analysis software. Coding and analysis were guided by LaRossa's application of grounded theory (41). This process of inductive qualitative analysis begins with *open coding*, or a line-by-line examination of interview designed to identify broad concepts derived from the data. While original conceptualizations of grounded theory discouraged the use of apriori constructs through the coding and analytic process, more recent interpretations incorporate the use of *sensitizing concepts* to guide preliminary coding of the data. Sensitizing concepts are described as “interpretive devices” that provide an organizing framework for making sense of the data (42). Often informed by the investigators' basic research questions, sensitizing concepts guiding this study's initial coding included patients' communication preferences, the roles adopted by both patients and providers, and patients' attitudes toward decisional authority.

Another core component of LaRossa's approach to inductive analysis is the use of a constant comparative method, where similarities and differences between and within codes are examined iteratively throughout the analytic process. To accomplish this, two authors (EBM & DG) first independently coded a sub-sample of interviews, then met to compare codes, identify and refine key, emergent themes, and resolve any discrepancies through consensus building. A third research team member (YZI) not involved in the coding process also joined these meetings in order to triangulate data and enhance the objectivity of the coding process. The initial codebook was iteratively refined as codes were adapted, changed, or distilled into more precise constructs. An audit trail of all team meetings and codebook revisions was kept to promote rigor. A total of 20% of transcripts (*n* = 6) were co-coded in this manner in order to establish reliability in coding and ensure no new themes emerged. Once the research team agreed that saturation had occurred, two research team members (EBM & DG) then independently coded the remaining transcripts.

Inductive, thematic analysis (43) was used in order to address key research questions about how decisions about depressions treatment were made in primary care, and how patients describe their own communication preferences relating to their care. To support this inductive analysis, the authors also used memo writing (44) as a strategy to both clarify and make meaning of emergent themes relating to these processes. Cross-case analysis (45) was also used to examine differences among the diverse sample. Additional strategies to increase the trustworthiness of the themes identified in this data included routine peer debriefing, triangulation, an audit trail, and negative case analysis (46).

## Results

### Study Sample

Study recruitment continued until the research team achieved a consensus that thematic saturation (47) was reached, meaning that no new or distinct codes or themes were evident from interviews. Of the 314 individuals successfully contacted for enrollment, 226 declined participation (i.e., did not want to enroll), 59 were lost to follow up after an initial contact, and 29 individuals were successfully enrolled. Of those who participated in the study, technical issues compromised the quality of two participants' responses, for a total sample of 27 patient respondents. Of the sample, the majority (70%, *n* = 19) were female. Over half (55%, *n* = 15) identified as Black or African American, while 33% (*n* = 3) identified as non-Hispanic white, 3.7% (*n*– = 1) were non-white Hispanic/Latinx, (*n* = 1) and 3.7% (*n* = 1) identified as “other” or unspecified. In addition, over half of the sample (51.8%, *n* = 14) had a college degree or more, 15% (*n* = 4) reported some college, and a third of the sample (*n* = 9) with a high school education or less. Finally, a most respondents fell into a 18–35 year old age bracket (33%, *n* = 9) or a 46–55 year old age bracket (33%, *n* = 9). Of the remaining sample, about 19% (*n* = 5) were between the ages of 36–45, and 11% (*n* = 3) were over 65 years of age. The vast majority of respondents (82%, *n* = 22) were engaged in some form of depression treatment at the time of interview. Among those receiving some form of care, ~55% of patients (*n* = 15) were taking antidepressants and 55% (*n* = 15) were in engaged in counseling or therapy, with 25% (*n* = 8) receiving both medication and therapy.

### Patient Experiences of Shared Decision-Making

Respondents reported relatively high levels of SDM, with an average score of 68 (SD = 26.2) on a 0–100 point scale. Results from qualitative interviews also suggested that patients within this sample experienced high levels of SDM and were satisfied with their ability to communicate effectively with their provider, as reflected in these responses:

“*Um so my doctor [name], can't recommend him like incredible, incredible, incredible, I‘ve never felt that comfortable with a doctor.”*

“*So, it was really, really good, umm like a trusting relationship. I also say umm he helps me solidify the conflict that I had.”*

“*Yeah, I feel like I've been blessed with that (doctor) is open to any questions. And it's never, she never makes me feel like, okay you are taking a little bit too much of my time. She's always, when I began to ask her something that I was like no no. You ask me what you need, she insists that I ask a question.”*

### Preferences for Communication and Decision Making

Building upon these largely positive experiences with their providers, respondents were able to identify several conditions or behaviors that articulated their preferred methods of communication and decision making, including factors that facilitated or inhibited shared decision-making practices.

#### Stigma Shaping Communication Preferences

Respondents described an acute awareness of the stigma associated with mental illness, particularly when they initially presented for care:

“*Well the stigma that people have about people suffering from mental health issues you know it's not easy though. It's not, to put yourself out there.”*

This perceived stigma appeared to motivate some of the communication and decision-making preferences described by respondents in the following sections. Because of existing, negative associations with depression or mental illness, many respondents described being “anti-medication” when initially presenting for depression treatment. Common concerns included negative side effects from antidepressants, and the stigma associated with being medicated for a mental health condition. When first engaging with providers about their depression, respondents also described an expectation that providers would adopt a paternalistic approach to decision-making, specifically one that would pressure patients to take medication. Together, this meant that many respondents initiated treatment discussions with the expectation that their own preferences (against medication) would be at odds with the preferences of their providers (advocates of medication). As a consequence, they presented to treatment with the perceived need to proactively protect against violations to their autonomy, including being coerced into taking psychiatric medication. Within the context of decision-making preferences, these underlying attitudes seemed to motivate participants' strong desire to maintain decisional authority, as described in the following sections.

#### Preferences for Decisional Autonomy

Respondents often described decision-making, including SDM, interchangeably in terms of an *outcome* (i.e., who makes a decision) and a *process* (i.e., how information is exchanged during the communication leading up to a decision). When initially asked how decisions about their depression care were made, respondents overwhelming remarked that they controlled decisions about their treatment:

“*I did the research, I brought up the concern, I made the final call this is what I wanted.”*

“*it was totally my decision. Yeah, it's totally my decision.”*

While these responses suggested a consistent preference for patient-led decision making, further examination indicated that this preference primarily applied to the decisional *outcome*, or who made the ultimate choice about depression treatment. Patients' persistent preference appeared related to their concern about being coerced or forced into treatment by their provider. Respondents were clear in their intent to disengage from providers who undermined their control over the decisional outcome:

“*I mean I've had those issues with other physicians where I felt like the attitude was like look just take what I told you to don't ask questions. I you know, I've gotten that response from some. And um, I don't want a doctor like that”*

In this way, respondents signaled a clear aversion to purely authoritarian, provider-led approaches to shaping treatment, and also underscored patients' tendency to protect against coercive practices by asserting their final right to approval or decline care. One respondent explained the importance of decisional control in this way:

“*it puts a lot of control on my hands and I feel like depression is about feeling like you're helpless a lot of the times. So when you see you have control over something it really helps out.”*

Despite these clear preferences, the *process* of information exchange leading up to the decision-making reflected a different type of expectation from patients. Respondents depended on providers to share their expertise about medication options and medication side effects in a way that was accessible and understandable. In addition, respondents actively sought out their providers' opinions or advice about which medication or treatment option would be optimal, and factored this into their own determinations of appropriateness. Several respondents described such a process in this way:

“*just about every decision that I've made with him as my doctor, has felt like I was making the decision he was confirming that it was like a good safe decision and then would sort of we would make like a plan to go from there.”*

“*there is a reason that I go to my healthcare provider. Which is because they have expertise so. You know if I, example let's say that I decided to go back on medication tomorrow. I wouldn't march into my doctor's office and demand a prescription. For specific medication that I wanted, Right I will chat with them about what they thought were my options or the benefits or a drawback of each one.”*

“*So, I feel always involved but I also feel like [doctor] wouldn't let me do something that he didn't think it was a good idea.”*

Rather than a patient directed process, these discussions were described as a partnership between the doctor and the provider, meaning that both parties shared information, articulated preferences or recommendations, and came to an agreed path forward. As the above quotes suggest, as part of this process respondents actively sought validation from their provider that their chosen treatment option was a good one, and often took heed of their recommendations. This reflects both a simultaneous need to feel control over the decision-outcome, while also desiring providers to offer expert guidance to help them navigate their treatment options. This mutual collaboration contrasts with respondents' firm assertion of their decisional authority described above, suggesting that patients' preferences related to the *process* and *outcome* of a shared decision-making process may be different.

#### Preference for Person-Centered Care, Rather than Depression-Centered Care

Respondents were attuned to signals that providers viewed them as “more than a diagnosis,” and sought an authentic, interpersonal connection with their doctor. As one respondent described it:

“*Yeah, it makes me feel like I matter to them. You know, I'm just not a paying customer. But, genuinely care about your health and what these meds do to you, that's always comforting to me.”*

Respondents experienced this genuine concern through several discrete actions, including providers' updated knowledge on their full medical history, and by their interest and understanding of life outside of depression, including patients' interests, responsibilities, and overall preferences toward their treatment. When providers effectively conveyed their understanding of respondents within the unique context of their unique life early on in treatment, respondents perceived them as better equipped to guide them during subsequent decision-making processes. Specifically, as respondents described, when providers understood the client as a person, they were better able to tailor psychoeducation and information giving to the particular preferences of the patient. The assurance that providers both valued and understood the respondent as a person ultimately seemed to increase the likelihood that they would be receptive to providers' advice or recommendations, as illustrated here:

“*She'll say ‘you were on this medicine before “and I will say “I don”t know why I went off of it” and she'll say “well-this one conflicted with your other drug” and that 1–10 years ago I was on why did I ever go off of that: “I think because weight gain was the side effect maybe sexual side effects or something like that” so we like we were pretty good together about piecing it out and seeing what the pros and cons of each drug are so that is really helpful…Yea and being really honest you know being really honest with yourself to someone else is kind of hard but she's got my back so..”*

#### Sharing the Role of Expert

Because retaining control of the decisional outcome was important to respondents, they also looked for indications that providers would be willing to share the role of the expert. Within the context of depression care, respondents were particularly sensitive to the experience of being believed when disclosing their experience of symptoms, including when their medication was not working properly, when they felt a particular treatment option was not ideal for them, or when their symptoms were fluctuating. Further, respondents described that the strongest partnerships occurred when providers centered treatment decisions around improving patients' subjective experience and quality of life, rather than symptom reduction alone. Both of these experiences are illustrated here:

“*So, he would frequently check in during when I'm still on [medication] and make sure that my dosage was correct. One point I did go up um and every time I went in it wasn't just a discussion of like “everything is good, right?” It would be like, “could this be better?”'*

“*[the doctor] felt very open to allow me to, you know, see if there is any problems [with medication] or allowed me to see what would be best at this time. And whenever I didn't feel it was right he let me up it or lower it my rate.”*

As one respondent noted, when providers demonstrated a receptiveness to patients' subjective experience of wellness and well-being, this increased the likelihood that patients would readily disclose issues with medication or changes to their depressive symptoms:

“*The [doctor] really tooked his time umm and would just like listen to me when I went, and I feel like. I was a lot more honest about the symptoms I was having the more I started to see him. Just because I feel a lot more comfortable with him.”*

#### Trust as a Preceding Condition to SDM

In order to create their preferred type of doctor-patient partnership already described, respondents acknowledged that they needed to be honest and transparent about the symptoms they were experiencing. In order to disclose openly in this way, respondents consistently underscored the need for an established sense of safety and trust with their provider:

“*I tried to look for something that really give me um, that I feel safe. That I feel safe cause I don't like to talk about this too much or I don't know sometimes I feel people will don't understand how I feel.”*

Nearly all respondents in this sample described having a trusting relationship with their current provider. During the decision-making process, respondents described that providers would often offer advice or recommendations about treatment options. Whether such advice was perceived as prescriptive and authoritative or helpful suggestions seemed to depend greatly on the degree trust and safety that existed between patients and providers.

#### Importance of Continuity of Care

Although conversations were intended to explore the process of decisions about depression were made, respondents consistently described the importance of proactive continuity of care *after* decisions occurred. Many patients in this sample had a long history of depression treatment, and an established relationship with their provider. From this perspective, patients emphasized the importance of accessibility:

“*This is why the rates of suicide is, everything happens the way it is because doctors allow their patients to fall through the crack.”*

Respondents reflected that decisions around depression care are not singular, and many articulated how their treatment needs and preferences change over time. Because of this, patients emphasized the need for providers to be available when they were needed, and for providers to take proactive approach to following up to ensure continuity of care:

“*having a doctor that will call and check up on you and that when he sees a form with my name on it. And he sees that I am going in and doing something.”*

“*he would try to you know make sure that I'd schedule meetings with him to check in, you know every few months just to see how everything was going on so he's very involved.”*

As above, patients described these gestures as genuine investments in in their continued well-being, and also appeared to function as continued reassurance that they would not lose their ability to initiate conversations about changing or terminating their depression treatment if needed.

## Discussion

The present study reflects upon the experiences of SDM and decision-making preferences among underserved primary care patients with depression. Overall, respondents described positive experiences of decision-making during depression care, which contrasts with findings from previous literature suggesting that SDM practices are not being readily infused into discussions about depression in primary care (28, 29, 31). These findings offer meaningful and needed insight into how underserved populations define preferred process of SDM in primary-care based depression treatment, and can guide future adaptation of SDM practices to align with the needs of underserved populations.

One notable theme was the prominent influence of stigma associated with depression and its impact on respondents' sensitivity toward being judged or labeled by their providers. The increased sensitivity influenced patients' initial openness toward depression treatment and their attitudes toward discussing symptoms with their provider. This finding is consistent with the wider literature, which has suggested that in the US, individuals from racial and ethnic minority groups are more likely to perceive stigma associated with mental illness than non-Hispanic whites, and, as a consequence, may be less likely to seek treatment for depressive symptoms (48, 49) or have negative attitudes toward depression treatment (50).

Results from this study contribute to this existing work by illustrating how perceived stigma informed respondents' preferences around communication and decision-making. First, although respondents generally described a preferences for collaborative communication during the decision making process, they also indicated a strong preference to retain control over the decisional outcome of depression treatment, specifically the authority to decline or accept services to treat their symptoms. This assurance appeared motivated by a perceived need to guard against coercive practices. Second, respondents emphasized that stigma could prevent effective SDM by inhibiting open and honest communication with providers, or engendering mistrust of providers' recommendations. Consequently, among this sample, successful SDM was dependent upon the development of a safe and non-judgmental environment.

In describing their preferences around communication and decision-making practices, respondents identified several provider behaviors that helped facilitate an environment where effective SDM could occur. Importantly, the behaviors that respondents emphasized most often, namely the development of trust, and consistent, reliable follow-up after making treatment decisions, occurred either *before* or *after* the decision-making process itself. While the importance of developing a strong working alliance is well-established in the mental health literature (51) leading models of SDM tend to approach decision-making as an isolated clinical process. In a critique of such SDM frameworks, Matthias et al. (52) have suggested that decision-making practices are inherently shaped by the overall relationship between the patient and provider, and therefore cannot be divorced from the larger clinical context. Findings from this study underscore this perspective, and point to the need to better account for interpersonal dynamics when implementing SDM in practice, especially with underserved communities, where differences in power and privilege may be particularly acute (53).

While adaptability has been identified as a necessary, albeit complex, component for effective dissemination of best practices (54), research around SDM has been slower to offer refined frameworks, or develop mechanisms that support successful implementation of SDM across diverse contexts (17, 52, 55, 56). To cultivate this environment, providers should first be aware of how the experience of stigma can inhibit patients' disclosure of symptoms, and focus early efforts on signaling their willingness to share the role of the expert with patients and establishing a safe and nonjudgmental space for patients. Respondents in this study suggest that particular components of effective practice include prioritizing and responding to patients' subjective well-being (including negative responses to medication), and maintaining a working knowledge of patients' full range of health and mental health needs.

There are several limitations to this study. First, while these findings provide an in-depth description of patient preferences for SDM, results from this study are not generalizable, and the preferences described by sample population may not be representative of all patients receiving depression treatment in primary care. Second, although the study took place within an urban, safety net ambulatory setting, the respondents in this sample were generally well-educated, and described a substantial history of receiving treatment for their depression. Because of this, this group may not reflect the needs of the most vulnerable population, including those with low health and mental health literacy or those considering depression treatment for the first time. However, respondents' robust historical experiences allowed them to provide rich detail about their challenges discussing treatment and disclosing symptoms, and reflect on the difficulties of negotiating stigma and power imbalances between patients and providers. Further, while about half of this sample had a college education and may therefore reflect those with higher educational attainment, it was quite diverse in terms of age and racial/ethnic background. A majority of literature around SDM reflects a predominantly white sample population, and as such this work fills an important gap in the representation of diverse patient groups in the literature. In addition, because respondents were reflecting on past experiences in care there is a risk for recall bias, and participants may be receiving depression care from multiple providers, including physicians, psychiatrists and therapists. Because all respondents had an active diagnosis of depression and the vast majority (80%) were actively receiving treatment, we believe this risk should be minimal. Additionally, respondents were prompted to speak specifically about their conversations about depression with their primary care provider during the interview process. Finally, this study took place during the COVID-19 pandemic, which required us to do all recruitment and data collection over the phone and via video conferencing. This may have impacted the composition of this sample, as those with greater accessibility to technology were more easily able to participate. Despite this, the success accessing a diverse sample demonstrates that conducting in-depth interviews virtually is feasible.

In sum, this study offers novel, in-depth insight into how patients prefer to approach discussions around depression treatment, highlighting areas where both decisional authority and partnership is desired (57). Through these in-depth interviews, respondents highlighted several strategies that can inform providers' efforts to encourage patient's to actively engage in conversations about depression treatment, and guide flexible and responsive applications of SDM that are most aligned with the needs of individuals seeking depression care.

## Data Availability Statement

The datasets presented in this article are not readily available because data includes protected patient information. Requests to access the datasets should be directed to ematthews13@fordham.edu.

## Ethics Statement

The studies involving human participants were reviewed and approved by Temple University Institutional Review Board. The patients/participants provided their written informed consent to participate in this study.

## Author Contributions

EM and YZ-I were primarily responsible for the study design and manuscript preparation for this project. MS and AP also informed the study design and assisted with recruitment. DW, TH, and DG assisted with primary data collection. EM, DG, and YZ-I all contributed to the analysis of the data presented. All authors contributed to the article and approved the submitted version.

## Conflict of Interest

The authors declare that the research was conducted in the absence of any commercial or financial relationships that could be construed as a potential conflict of interest.
